# Population attributable fractions for modifiable risk factors of neonatal, infant, and under-five mortality in 48 low- and middle-income countries

**DOI:** 10.7189/jogh.15.04015

**Published:** 2025-01-17

**Authors:** Kedir Y Ahmed, Subash Thapa, Getiye D Kibret, Habtamu M Bizuayehu, Jing Sun, M Mamun Huda, Abel F Dadi, Felix A Ogbo, Shakeel Mahmood, Muhammad J. A. Shiddiky, Fentaw T Berhe, Setognal B Aychiluhm, Anayochukwu E Anyasodor, Allen G Ross

**Affiliations:** 1 Rural Health Research Institute, Charles Sturt University, Orange, New South Wales, Australia; 2 Faculty of Medicine, Health and Human Sciences, Macquarie University, Macquarie Park, New South Wales, Australia.; 3 First Nations Cancer and Wellbeing (FNCW) Research Program, School of Public Health, The University of Queensland, St Lucia, Queensland, Australia; 4 Data Science Institute, University of Technology Sydney, Sydney, New South Wales, Australia; 5 School of Health Sciences and Social Work, Griffith University, Gold Coast, Queensland, Australia; 6 Menzies School of Health Research, Charles Darwin University, Casuarina, Northern Territory, Australia; 7 Addis Continental Institute of Public Health, Addis Ababa, Ethiopia; 8 Riverland Academy of Clinical Excellence (RACE), Riverland Mallee Coorong Local Health Network, SA Health Government of South Australia, Berri, South Australia, Australia; 9 Translational Health Research Institute, Western Sydney University, Campbelltown, New South Wales, Australia; 10 Department of Epidemiology and Biostatistics, School of Public Health, College of Medicine and Health Sciences, Wollo University, Dessie, Ethiopia; 11 School of Medicine and Dentistry, Griffith University, Gold Coast, Queensland, Australia

## Abstract

**Background:**

Identifying the modifiable risk factors for childhood mortality using population-attributable fractions (PAFs) estimates can inform public health planning and resource allocation in low- and middle-income countries (LMICs). We estimated PAFs for key population-level modifiable risk factors of neonatal, infant, and under-five mortality in LMICs.

**Methods:**

We used the most recent Demographic and Health Survey data sets (2010–22) from 48 LMICs, encompassing 35 sub-Saharan African countries and 13 countries from South and Southeast Asia (n = 506 989). We used generalised linear latent mixed models to compute odds ratios (ORs), and we calculated the PAFs adjusted for commonality using ORs and prevalence estimates for key modifiable risk factors.

**Results:**

The highest PAFs of neonatal mortality were attributed to delayed initiation of breastfeeding (>1 hour of birth) (PAF = 23.9; 95% confidence interval (CI) = 23.1, 24.8), uncleaned cooking fuel (PAF = 6.2; 95% CI = 6.4, 7.8), infrequent antenatal care (ANC) visits (PAF = 4.3; 95% CI = 3.3, 5.9), maternal lack of formal education (PAF = 3.9; 95% CI = 2.7, 5.3), and mother’s lacking two doses of tetanus injections (PAF = 3.0; 95% CI = 1.9, 3.9). These five modifiable risk factors contributed to 41.4% (95% CI = 35.6, 47.0) of neonatal deaths in the 48 LMICs. Similarly, a combination of these five risk factors contributed to 40.5% of infant deaths. Further, delayed initiation of breastfeeding (PAF = 15.8; 95% CI = 15.2, 16.2), unclean cooking fuel (PAF = 9.6; 95% CI = 8.4, 10.7), mothers lacking formal education (PAF = 7.9; 95% CI = 7.0, 8.9), infrequent ANC visits (PAF = 4.0; 95% CI = 3.3, 4.7), and poor toilet facilities (PAF = 3.4; 95% CI = 2.6, 4.3) were attributed to 40.8% (95% CI = 36.4, 45.2) of under-five deaths.

**Conclusions:**

Given the current global economic climate, policymakers should prioritise these modifiable risk factors. Key recommendations include ensuring that women enter pregnancy in optimal health, prioritising the presence of skilled newborn attendants for timely and proper breastfeeding initiation, and enhancing home-based care during the postnatal period and beyond.

The Sustainable Development Goals (SDGs) target 3.2 aims to reduce neonatal mortality to as low as 12 per 1000 live births and under-five mortality to as low as 25 per 1000 live births by 2030 [1]. Despite these targets, an estimated five million children aged <5 years lost their lives worldwide in 2021, with over 80% (4.1 million) of these deaths occurring in just two regions – sub-Saharan Africa (SSA) and South Asia [2]. With no additional health care investments, nearly 40 million children are projected to lose their lives before 2030, putting many countries in SSA and South Asia at risk of falling short of meeting the SDG targets for neonatal and under-five mortality [3].

Many low- and middle-income countries (LMICs) have made substantial progress in reducing under-five mortality rates over the past three decades [2]. LMICs, including Ethiopia, Malawi, and Uganda in SSA and Bangladesh, Mongolia, and Uzbekistan in Asia, have successfully exceeded the 59% global decline in under-five mortality by achieving reductions of over 75% between 1990–2021 [2]. This success highlights the feasibility of reducing child mortality and underscores its achievability across countries with diverse socio-economic and political contexts.

The improved socioeconomic conditions in some of these countries have naturally contributed to the decrease in under-five mortality rates [4,5]. However, several LMICs still face challenges due to slow-developing economies and political landscapes [6]. These difficulties extend to inadequate public health and health care responses, resulting from inadequate prioritisation and resource allocation to improve the health of the mother and child. To effectively address the persistent burden of under-five mortality in LMICs, policymakers and public health experts should have access to crucial information on modifiable risk factors at the population level. This information can guide targeted resource allocation and prioritising public health interventions.

Several studies in SSA and Soth Asia have examined sociodemographic, health service, and behavioural risk factors associated with childhood mortality using relative measures of association (e.g. odds ratios (ORs), relative risk (RR), and hazard risks) [7–11]. However, exclusive reliance on these relative measures, as indicated by ORs and RR, might not be the most efficient approach for appropriate public health planning and resource allocation [12,13]. For instance, a strong association between a risk factor and an outcome may have minimal public health importance if the factor is rare, while an association could have a more significant impact if the factor is more common [14].

To address this gap, several earlier studies have advocated for using population-attributable fraction (PAF) estimates to provide a more comprehensive understanding of the impact of modifiable risk factors [14,15]. The PAF methodology estimates the proportion of cases that could be prevented if the risk factor were eliminated, offering a more actionable approach to public health planning [12,13,16]. PAFs integrate both the relative risk (‘seriousness’ of a risk factor) and the prevalence of the risk factor (frequency and level of exposure) in a particular population, allowing for an assessment of what risk factors are most important for population health. In resource-limited settings, where policymakers must make difficult decisions about allocating scarce resources, PAF is critical in translating epidemiological findings into effective policy interventions.

Accordingly, in this study, we aimed to determine the key population-level modifiable risk factors for neonatal, infant, and under-five mortality in LMICs and to what extent these mortalities can be reduced by addressing these risk factors. The findings from this study would be instrumental in informing the policy development targeting the reduction of the burden of childhood deaths in LMICs and achieving the SDGs target 3.2 by 2030 [1].

## METHODS

### Data sources

We used the nationally representative Demographic and Health Survey (DHS) data sets to estimate the PAFs for modifiable risk factors associated with neonatal, infant, and under-five mortality in 48 LMICs. Over the past three decades, DHS has been conducted in more than 90 LMICs across Africa, Asia, Latin America and the Caribbean, and Eastern Europe to facilitate informed public health decisions by providing accurate data on population, health and nutrition [17]. Health ministries or governmental agencies in each country conduct DHS surveys, with technical assistance provided by Inner-City Fund International, funded by the United States Agency for International Development.

In this study, we focused on 48 LMICs, including 35 SSA and 13 South and Southeast Asia (SSEA) countries. We selected these two regions based on their substantial contribution to global under-five and neonatal mortality [2] and data availability (Table S1 in the **Online Supplementary Document**). We followed the Strengthening the Reporting of Observational Studies in Epidemiology (STROBE) reporting guideline for cross-sectional studies.

### Sampling procedures and sample size

The DHS uses a two-stage stratified cluster sampling method to recruit the study participants. In the initial stage, the first administrative units (such as states and regions) were categorised into urban or rural strata, and enumeration areas (EAs) were then selected proportionally to the population size of each urban/rural stratum. A complete household census was conducted in each selected EA. In the second stage, a fixed number of households were chosen using the household list as a sampling frame.

Data were collected from eligible women, encompassing all females aged 15–49 years residing permanently in households or present on the night before the survey. For this study, we pooled the latest available DHS survey data sets (2010–22) from 48 SSA and SSEA countries (Table S1 in the **Online Supplementary Document**). The study involved a weighted sample of 506 989 singleton live births that occurred in the five years before each country survey across the 48 countries [18]. Our analyses were restricted to the youngest live births to minimise recall bias.

### Outcome variables

The main outcomes of this study included neonatal, infant and under-five mortality, calculated as deaths within specific age groups among live births in the five years preceding the survey. We calculated neonatal mortality as deaths within the first month of life, infant mortality deaths before the first birthday, and under-five mortality deaths before the fifth birthday. A death within the specified periods was coded as ‘1’ while no death was coded as ‘0.’ All mortality rates were expressed per 1000 live births.

### Modifiable risk factors

We broadly grouped the modifiable risk factors as child, maternal, and household factors. Child factors included perceived baby birth size and early initiation of breastfeeding. Maternal factors encompassed maternal education, antenatal care (ANC) visits, place of birth, and maternal tetanus toxoid vaccination. Household factors included wealth index, toilet facility, source of drinking water, and type of cooking fuel. The modifiable factors were selected based on past literature [19–22], their importance for the outcomes, availability of data, and the amenability for policy interventions in improving child health and survival. (**Table 1**; Appendix S2 in the **Online Supplementary Document**).

**Table 1:** Definitions for modifiable risk factors of neonatal, infant, and under-five mortality

Modifiable risk factors	Definitions
Child factors	
*Perceived birth size*	1 – below average birth size, 2 – average and above birth size
*EIBF*	1 – initiated breastfeeding within 1 h of birth, 2 – not initiated breastfeeding within 1 h of birth
Maternal factors	
*Maternal education*	1 – no or low schooling, 2 – secondary education or higher
*Maternal employment*	Not working or working
*Frequency of ANC visits*	1 – no or low ANC visits, 2 –≥4 visits
*Place of birth*	Home or health facility birth
*Maternal tetanus toxoid vaccination*	1 –<2 doses, 2 –≥2 doses
Household factors	
*Household wealth index*	1 – poor or medium households, 2 – rich households
*Source of drinking water and type of toilet facility*	1 – improved, 2 – not improved
*Type of cooking fuel*	1 – cleaned, 2 – not cleaned

ANC – antenatal care, EIBF – early initiation of breastfeeding

### Potential covariates

We considered the following potential covariates: the sex of the child (grouped as ‘female’ or ‘male’), the child’s birth order (grouped as ‘first child,’ ‘2 to <5 births,’ or ‘≥5 births’), maternal age (grouped as ‘15–24 years,’ ‘25–34 years,’ or ‘35–49 years’), family size (grouped as ‘2–3 members,’ ‘4–5 members,’ ‘+6 members’) and place of residence (grouped as ‘rural’ or ‘urban’).

### Statistical analysis

We initially calculated frequencies and percentages to present an overview of the study population. We also calculated estimates for neonatal, infant, and under-five mortality. Subsequently, we used generalised linear latent and mixed models in the pooled data to compute ORs with 95% confidence intervals (CIs) for the modifiable risk factors associated with neonatal, infant and under-five mortalities. The decision to use ORs in this study was based on the rarity assumption (prevalence <10%) observed in cases of neonatal, infant, and under-five mortalities, where there is a tendency for ORs and RRs to converge [23].

We implemented our generalised linear latent and mixed models in two levels, individual (*e.g.* child, maternal, and household factors) and community levels (place of residence), to account for the hierarchical nature of the data, wherein children aged <5 years were nested within geographic clusters. Multilevel models offer distinct advantages compared to classical single-level logistic regression models. First, it acknowledges the hierarchical nature of data, recognising that children aged <5 years (Level I) are nested within clusters (Level II). Failure to account for this hierarchy results in underestimated standard errors of regression coefficients, leading to an overstatement of statistical significance [24,25]. Second, multilevel modelling addresses the dependence of observations within the same clusters; children within the same cluster tend to be more similar than those in different clusters [24,25]. Lastly, it allows for the simultaneous estimation of cluster-level effects (random effects) and the assessment of associations for community-level predictors, such as place of residence.

We constructed the multilevel models in three steps. Initially, we developed a null unconditional model in stage one without any study variable. In stage two, we incorporated individual-level factors (including child, maternal, and household factors) into the model. In stage three, we introduced community-level factors and presented the results, which encompassed both individual and community-level factors. This final model, which included individual and community-level factors, was chosen due to its minimal deviance and superior ability to explain the variation in the outcome variables. Detailed statistical analyses (including assumption checks) procedures are provided in Appendix S3 in the **Online Supplementary Document**.

Upon identifying the modifiable risk factors associated with neonatal, infant, and under-five mortalities using the generalised linear latent and mixed models, our analysis involved the computation of PAFs using Miettinen’s formula. We chose Miettinen’s formula based on several reasons. First, Miettinen’s formula is generally preferred when ORs are used to measure association, particularly in rare outcomes. Second, the formula incorporates the prevalence of exposure among cases (Pc), making it suitable when the overall population prevalence is not well-known. Third, Miettinen’s formula offers greater flexibility for adjusting confounders, particularly when using adjusted ORs from multivariable models [26]. The PAF serves as a metric indicating the proportion of childhood deaths in LMICs that could potentially be mitigated by addressing the identified modifiable risk factors within the population [26]. We calculated PAF using the following formula:



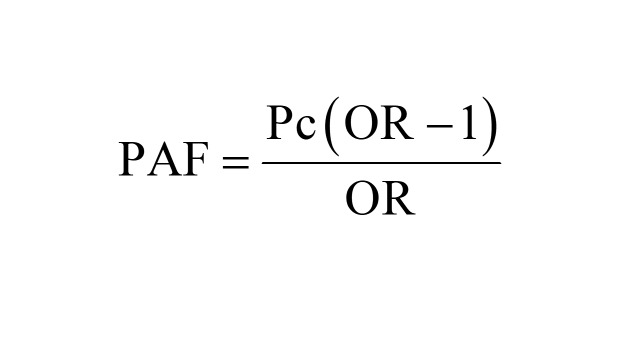



Where Pc is the prevalence of the modifiable risk factor among cases, and OR is the adjusted ORs of neonatal mortality associated with the modifiable risk factors [26]. Given the modifiable risk factors occur simultaneously within individuals, aggregating the PAF for each specific risk factor may lead to an overestimation of their combined effects [21,27]. Based on previously published studies [28], we employed communality weights to correct for the overlap of risk factors among participants [28].

To calculate the commonalities, we started by looking at how different modifiable risk factors are related using a tetrachoric correlation. This helps us understand how these risk factors interact and overlap within individuals. Next, we used a principal components analysis to identify the main patterns in the data. This analysis allows us to see which risk factors are most important and how they group. For each risk factor, we calculated its communality, which tells us how much of its variation is explained by these main patterns. Specifically, we did this by adding up the squares of the values (loadings) associated with each risk factor from the principal components with a significant impact (those with values >1). After calculating the communalities, we determined a weight for each risk factor using the formula: communality weight for each risk factor (We) = 1 − communality. This means that if a risk factor is highly explained by the common patterns (high communality), its weight will be lower, reflecting its shared influence with other factors.

Following this, we calculated a combined PAF across the modifiable risk factors using the specified formula:



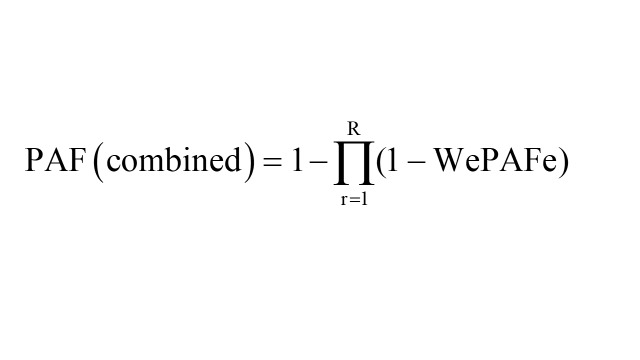



Where ‘e*’* represents each modifiable risk factor, PAFe represents PAF for each risk factor, and ‘We’ represents the communality weight of each risk factor. Finally, we estimated the adjusted PAF for each risk factor using the formula:



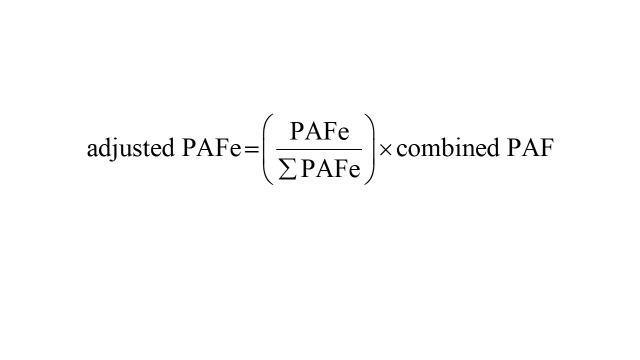



To address potential imbalances and unequal probabilities in household selections and non-responses and to account for clustering and stratification, we applied survey weighting to the data using the ‘svy’ command in STATA, version 18.0 (Stata Corp, College Station, TX, USA) [29]. We computed the neonatal, infant and under-five mortality rates using the ‘SYNCMRATES’ and regression analysis using the ‘GLLAMM’ package for STATA [30]. We present the association between the modifiable risk factors and the outcome variables in terms of ORs along with 95% CIs.

### Ethical considerations

Ethical approval was obtained for all DHS surveys from the relevant authorities in each country. In each DHS, verbal informed consent was obtained from participants (or their parents/guardians) before data collection began, ensuring privacy and confidentiality throughout the process. The DHS data set is anonymised and publicly accessible, containing no identifiable information about participants. Data access was granted by the Measure DHS following a formal request for permission to use the data.

## RESULTS

### Participants

This study included a weighted total sample of 506 989 under-five children with a mean age of 24.2 months (standard deviation = 16.3). Of these, 52.0% were males. Regarding maternal characteristics, 241 552 mothers (47.7%) attained secondary education or higher, and 149 102 (53.8%) were unemployed. Additionally, 383 740 mothers (78.0%) gave birth at a health facility, while 294 097 (62.1%) had less than two doses of tetanus vaccination during pregnancy. In terms of household characteristics, 190 511 children (37.6%) resided in rich households, and 164 490 (32.4%) were from urban households (**Table 2**).

**Table 2:** Characteristics of the study participants (n = 506 989)*

Variables	Male	Female	Total
EIBF			
*No*	135 722 (51.5)	123 272 (50.6)	258 994 (51.1)
*Yes*	127 829 (48.5)	120 167 (49.4)	247 996 (48.9)
Birth order			
*1*	71 665 (27.2)	66 096 (27.2)	137 761 (27.2)
*2–4*	141 445 (53.7)	128 831 (52.9)	270 276 (53.3)
*<5*	50 441 (19.1)	48 511 (19.9)	98 952 (19.5)
Maternal age in years			
*15–24*	79 490 (30.2)	73 837 (30.3)	153 327 (30.2)
*25–34*	135 169 (51.3)	122 285 (50.2)	257 454 (50.8)
*35–49*	48 892 (18.6)	47 317 (19.4)	96 208 (19.0)
Maternal education			
*No or low education*	136 762 (51.9)	128 657 (52.9)	265 419 (52.4)
*Secondary or higher*	126 778 (48.1)	114 774 (47.2)	241 552 (47.7)
Maternal employment			
*Not working*	76 797 (54.1)	72 306 (53.5)	149 102 (53.8)
*Working*	65 085 (45.9)	62 874 (46.5)	127 959 (46.2)
ANC visits			
*≤3*	104 495 (41.6)	96 292 (41.5)	200 787 (41.6)
*≥4*	146 755 (58.4)	135 592 (58.5)	282 346 (58.4)
Place of birth			
*Home*	54 951 (21.5)	53 021 (22.5)	107 972 (22.0)
*Health facility*	200 677 (78.5)	183 064 (77.5)	383 740 (78.0)
Maternal tetanus vaccination doses			
*<2*	153 918 (62.6)	140 180 (61.7)	294 097 (62.1)
*≥2*	92 116 (37.4)	87 208 (38.4)	179 324 (37.9)
Family size			
*2–3*	30 928 (11.8)	28 771 (11.8)	59 699 (11.8)
*4–5*	88 217 (33.6)	81 551 (33.6)	169 767 (33.6)
*>6*	143 831 (54.7)	132 632 (54.6)	276 464 (54.6)
Household wealth			
*Poor or medium households*	164 651 (62.5)	151 827 (62.4)	316 478 (62.4)
*Rich households*	98 899 (37.5)	91 612 (37.6)	190 511 (37.6)
Type of toilet system			
*Not improved*	103 619 (40.0)	97 051 (40.6)	200 670 (40.2)
*Improved*	155 699 (60.0)	142 300 (59.5)	297 999 (59.8)
Source of drinking water			
*Not protected*	96 531 (36.6)	90 169 (37.0)	186 700 (36.8)
*Protected*	167 020 (63.4)	153 269 (63.0)	320 289 (63.2)
Type of cooking fuel			
*Not cleaned*	93 313 (35.5)	83 992 (34.6)	177 305 (35.0)
*Cleaned*	169 744 (64.5)	15 8968 (65.4)	328 713 (65.0)
Place of residence			
*Urban*	84 947 (32.2)	79 543 (32.7)	164 490 (32.4)
*Rural*	178 604 (67.8)	163 896 (67.3)	342 500 (67.6)

ANC – antenatal care, EIBF – early initiation of breastfeeding

*Presented as n (%).

### Neonatal, infant and under-five mortality rates

**Figure 1:**
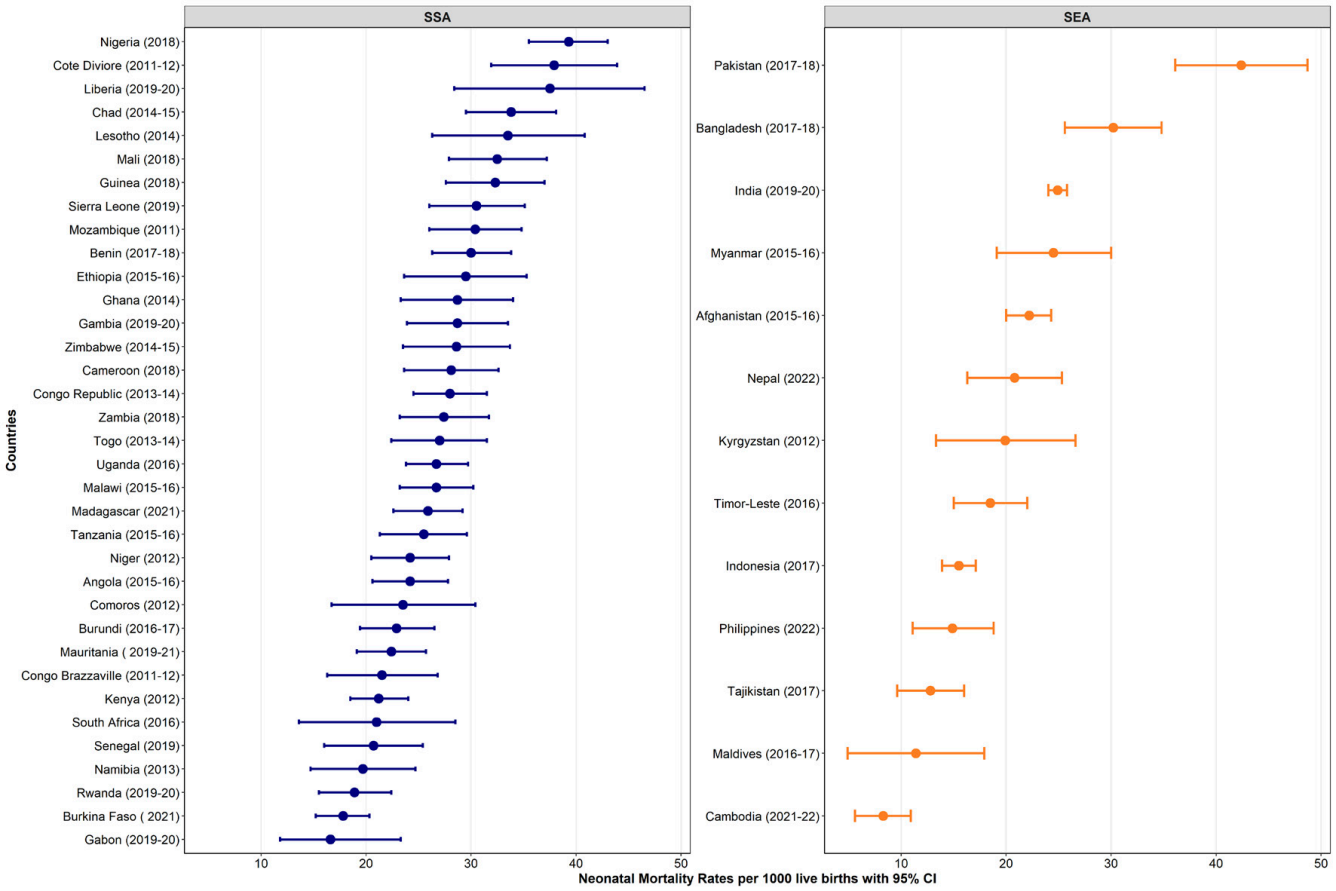
Neonatal mortality rates per 1000 across 48 LMICs. CI – confidence interval, SEA – Southeast Asia, SSA – sub-Saharan Africa.

**Figure 2:**
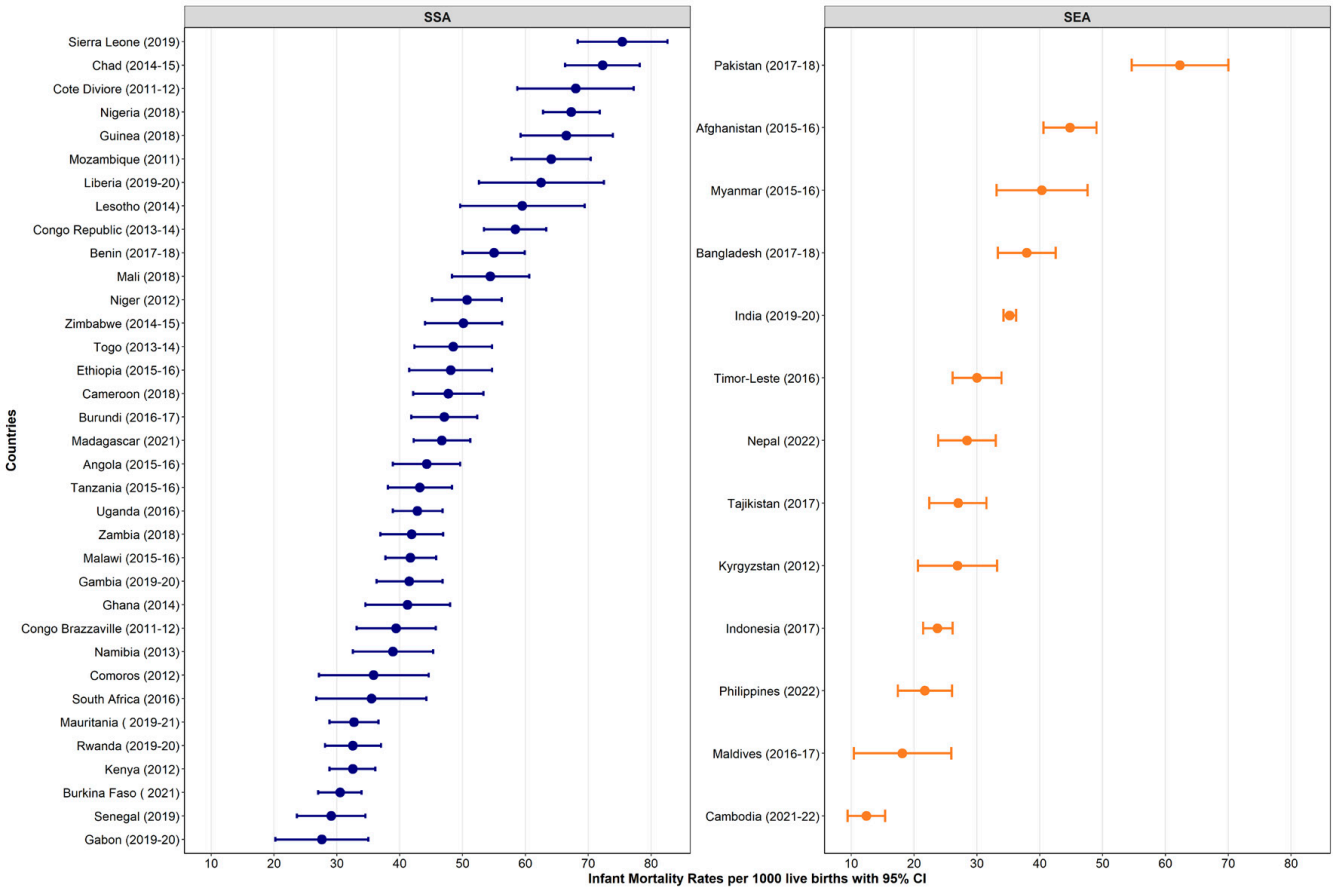
Infant mortality rates per 1000 across 48 LMICs. CI – confidence interval, SEA – Southeast Asia, SSA – sub-Saharan Africa.

**Figure 3:**
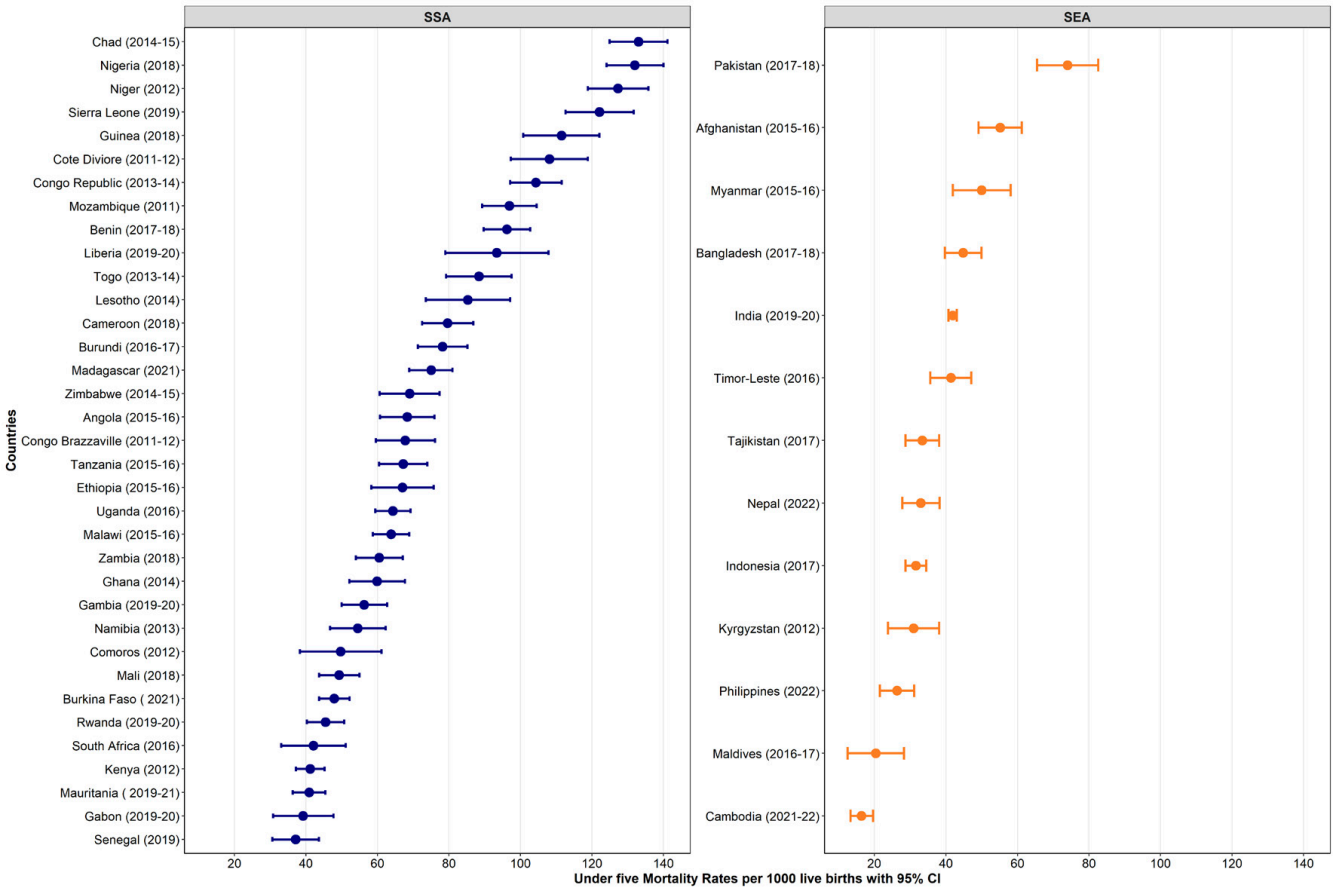
Under-five mortality rates per 1000 across 48 LMICs. CI – confidence interval, SEA – Southeast Asia, SSA – sub-Saharan Africa.

The analysis of neonatal mortality rate (NMR), infant mortality rate (IMR), and under-five mortality rate revealed variations across countries. Pakistan had the highest NMR (NMR = 42.4 per 1000 live births; 95% CI = 36.1, 48.7) in 2017–18, while Cambodia had the lowest (NMR = 8.3 per 1000 live births; 95% CI = 5.6, 10.9) in 2021–22 (**Figure 1**). Similarly, the highest IMR was observed in Sierra Leone (IMR = 75.4 per 1000 live births; 95% CI = 68.3, 82.6) in 2019, while Cambodia had the lowest (IMR = 12.4 per 1000 live births; 95% CI = 9.4, 15.4) in 2021–22 (**Figure 2**). The highest under-five mortality rate was found in Chad (under-five mortality rate = 133.0 per 1000 live births; 95% CI = 124.9, 141.1) in 2014–15, while the lowest was observed in Cambodia (under-five mortality rate = 16.4 per 1000; 95% CI = 13.3, 19.6) in 2021–22 (**Figure 3**).

### Population attributable fractions for neonatal mortality

To investigate the factors contributing to neonatal mortality, we calculated PAFs. The largest PAFs of neonatal mortality were associated with delayed initiation of breastfeeding (PAF = 23.87; 95% CI = 23.11, 24.79), use of uncleaned cooking fuel (PAF = 6.19; 95% CI = 4.61, 7.81), mothers not having frequent ANC visits (PAF = 4.27; 95% CI = 3.27, 5.94), mothers lacking formal education (PAF = 3.93; 95% CI = 1.92, 3.92), and less than two maternal tetanus injections before birth (PAF = 2.96; 95% CI = 1.92, 3.92) (**Table 3**). Collectively, these five modifiable risk factors were associated with 41.4% (95% CI = 35.6, 47.0) of neonatal deaths in LMICs.

**Table 3:** Population-attributable fractions for neonatal mortality in 48 LMICs, 2010–22

Variables	Prevalence of risk factors in cases (95% CI)	OR (95% CI)	Unweighted PAF% (95% CI)	Weighted PAF% (95% CI)*
EIBF				
*No*	78.45 (77.29, 79.57)	3.56 (3.38, 3.76)	56.45 (54.43, 58.43)	23.87 (23.11, 24.79)
*Yes*	21.55 (20.43, 22.71)	Ref.	Ref.	Ref.
Maternal education				
*No or low education*	60.46 (59.00, 61.91)	1.18 (1.12, 1.25)	9.23 (6.32, 12.38)	3.93 (2.68, 5.25)
*Secondary or higher*	39.54 (38.09, 41.01)	Ref.	Ref.	Ref.
ANC visits				
*≤3*	51.84 (50.38, 53.31)	1.24 (1.18, 1.30)	10.03 (7.69, 12.30)	4.27 (3.27, 5.94)
*≥4*	48.16 (46.69, 49.62)	Ref.	Ref.	Ref.
Place of birth				
*Home*	26.25 (24.93, 27.61)	0.93 (0.88, 0.99)	–2.00 (–3.40, –0.28)	NA
*Health facility*	73.75 (73.39, 75.07)	Ref.	Ref.	
Maternal tetanus vaccination doses				
*<2*	56.49 (54.94, 58.03)	1.14 (1.09, 1.19)	6.94 (4.53, 9.26)	2.96 (1.92, 3.92)
*≥2*	43.51 (41.97, 45.06)	Ref.	Ref.	Ref.
Household wealth				
*Poor or medium households*	68.88 (67.45, 70.28)	1.02 (0.96, 1.08)	1.35 (–2.81, 5.21)	NA
*Rich households*	31.12 (29.72, 32.55)	Ref.	Ref.	
Type of toilet system				
*Not improved*	46.52 (45.01, 48.04)	1.03 (0.98, 1.09)	1.35 (–0.92, 3.96)	NA
*Improved*	53.48 (51.96, 54.99)	Ref.	Ref.	
Source of drinking water				
*Not protected*	38.06 (36.67, 39.48)	0.98 (0.93, 1.02)	–0.78 (–2.76, 0.77)	NA
*Protected*	61.94 (60.52, 63.33)	Ref.	Ref.	
Type of cooking fuel				
*Not cleaned*	72.72 (71.22, 74.17)	1.25 (1.18, 1.33)	14.54 (10.86, 18.41)	6.19 (4.61, 7.81)
*Cleaned*	27.28 (25.83, 28.78)	Ref.	Ref.	Ref.

ANC – antenatal care, CI – confidence interval, EIBF – early initiation of breastfeeding, NA – not applicable, OR – odds ratio, PAF – population attributable fraction, ref – reference

*Weighted PAF is the relative contribution of each risk factor to the overall PAF when adjusted for communality.

### Population attributable fractions for infant mortality

Similarly, we examined the PAFs for infant mortality. The largest PAFs of infant mortality were associated with delayed initiation of breastfeeding (PAF = 17.84; 95% CI = 17.05, 18.18), use of uncleaned cooking fuel (PAF = 8.05; 95% CI = 6.50, 9.23), mothers lacking formal education (PAF = 6.53; 95% CI = 5.54, 7.59), mothers not having frequent ANC visits (PAF = 4.11; 95% CI = 2.80, 4.75), and less than two maternal tetanus injections before birth (PAF = 3.74; 95% CI = 2.73, 4.50) (**Table 4**). These modifiable risk factors together were associated with 40.5% (95% CI = 35.2, 44.9) of infant deaths in LMICs.

**Table 4:** Population-attributable fractions for infant mortality in 48 LMICs, 2010–22

Variables	Prevalence of risk factors in cases (95% CI)	OR (95% CI)	Unweighted PAF% (95% CI)	Weighted PAF% (95% CI)*
EIBF				
*No*	69.28 (68.22, 70.32)	2.20 (2.12, 2.29)	37.80 (36.03, 39.60)	17.84 (17.05, 18.18)
*Yes*	30.72 (29.68, 31.78)	Ref.	Ref.	Ref.
Maternal education				
*No or low education*	63.85 (62.62, 65.07)	1.28 (1.23, 1.34)	13.98 (11.71, 16.52)	6.53 (5.54, 7.59)
*Secondary or higher*	36.15 (34.93, 37.38)	Ref.	Ref.	Ref.
ANC visits				
*≤3*	52.06 (50.85, 53.27)	1.22 (1.17, 1.26)	9.40 (7.40, 11.00)	4.11 (2.80, 4.75)
*≥4*	47.94 (46.73, 49.15)	Ref.	Ref.	Ref.
Place of birth				
*Home*	28.92 (27.76, 30.12)	1.07 (1.02, 1.11)	1.89 (0.55, 2.98)	NA
*Health facility*	71.08 (69.88, 72.24)	Ref.	Ref.	
Maternal tetanus vaccination doses				
*<2*	55.20 (53.93, 56.46)	1.17 (1.12, 1.21)	8.02 (5.78, 9.81)	3.74 (2.73, 4.50)
*≥2*	44.80 (43.54, 46.07)	Ref.	Ref.	Ref.
Household wealth				
*Poor or medium households*	69.83 (68.64, 70.99)	1.01 (0.96, 1.06)	0.69 (–2.86, 1.23)	NA
*Rich households*	30.2 (29.0, 31.4)	Ref.	Ref.	
Type of toilet system				
*Not improved*	49.40 (48.14, 50.66)	1.13 (1.09, 1.18)	5.68 (3.97, 7.73)	2.65 (1.88, 3.55)
*Improved*	50.60 (49.34, 51.86)	Ref.	Ref.	Ref.
Source of drinking water				
*Not protected*	39.66 (38.46, 40.88)	1.02 (0.98, 1.06)	0.78 (–0.79, 2.32)	NA
*Protected*	60.34 (59.12, 61.54)	Ref.	Ref.	
Type of cooking fuel				
*Not cleaned*	74.67 (73.38, 75.91)	1.30 (1.23, 1.36)	17.24 (13.73, 20.09)	8.05 (6.50, 9.23)
*Cleaned*	25.33 (24.09, 26.62)	Ref.	Ref.	Ref.

ANC – antenatal care, CI – confidence interval, EIBF – early initiation of breastfeeding, NA – not applicable, OR – odds ratio, PAF – population attributable fraction, ref – reference

*Weighted PAF is the relative contribution of each risk factor to the overall PAF when adjusted for communality.

### Population attributable fractions for under-five mortality

Finally, we assessed the PAFs for under-five mortality. The highest PAFs of under-five mortality were associated with delayed initiation of breastfeeding (PAF = 15.78; 95% CI = 15.24, 16.20), use of unclean cooking fuel (PAF = 9.60; 95% CI = 8.35, 10.66), mothers lacking formal education (PAF = 7.92; 95% CI = 7.01, 8.88), mothers not having frequent ANC visits (PAF = 4.02; 95% CI = 3.28, 4.69), and unimproved toilet facilities (PAF = 3.40; 95% CI = 2.63, 4.26) (**Table 5**). In total, these five modifiable risk factors were associated with 40.8% (95% CI = 36.4, 45.2) of under-five deaths in LMICs.

**Table 5:** Population-attributable fractions for under-five mortality in 48 LMICs

Variables	Prevalence of risk factors among cases (95% CI)	OR (95% CI)	Unweighted PAF% (95% CI)	Weighted PAF% (95% CI)*
EIBF				
*No*	66.48 (65.46, 67.48)	1.96 (1.89, 2.03)	32.57 (30.84, 34.25)	15.78 (15.24, 16.2)
*Yes*	33.52 (32.52, 34.54)	Ref.	Ref.	Ref.
Maternal education				
*No or low education*	65.92 (64.77, 67.04)	1.33 (1.28, 1.39)	16.35 (14.18, 18.80)	7.92 (7.01, 8.88)
*Secondary or higher*	34.08 (32.96, 35.23)	Ref.	Ref.	Ref.
ANC visits				
*≤3*	51.94 (50.82, 53.05)	1.19 (1.15, 1.23)	8.29 (6.63, 9.93)	4.02 (3.28, 4.69)
*≥4*	48.06 (46.95, 49.18)	Ref.	Ref.	Ref.
Place of birth				
*Home*	28.90 (27.80, 30.10)	1.18 (1.13, 1.22)	4.41 (3.20, 5.43)	2.12 (1.58, 2.57)
*Health facility*	71.10 (67.00, 72.20)	Ref.	Ref.	Ref.
Maternal tetanus vaccination doses				
*<2*	54.87 (53.71, 56.02)	1.13 (1.10, 1.17)	6.32 (4.88, 8.14)	3.06 (2.41, 3.85)
*≥2*	45.13 (43.98, 46.29)	Ref.	Ref.	Ref.
Household wealth				
*Poor or medium households*	70.02 (68.93, 71.10)	0.99 (0.95, 1.03)	–0.71 (–3.63, 2.00)	NA
*Rich households*	29.98 (28.90, 31.07)	Ref.	Ref.	
Type of toilet system				
*Not improved*	50.82 (49.65, 52.98)	1.16 (1.12, 1.21)	7.01 (5.33, 9.02)	3.40 (2.63, 4.26)
*Improved*	49.19 (48.02, 50.35)	Ref.	Ref.	Ref.
Source of drinking water				
*Not protected*	39.97 (38.85, 41.10)	1.02 (0.98, 1.05)	0.78 (–0.79, 1.96)	NA
*Protected*	60.03 (58.90, 61.15)	Ref.	Ref.	
Type of cooking fuel				
*Not cleaned*	76.42 (75.24, 77.55)	1.35 (1.29, 1.41)	19.81 (16.91, 22.56)	9.60 (8.35, 10.66)
*Cleaned*	23.58 (22.45, 24.76)	Ref.	Ref.	Ref.

ANC – antenatal care, CI – confidence interval, EIBF – early initiation of breastfeeding, NA – not applicable, OR – odds ratio, PAF – population attributable fraction, ref – reference

*Weighted PAF is the relative contribution of each risk factor to the overall PAF when adjusted for communality.

## DISCUSSION

To the best of our knowledge, this is the first study examining PAFs for key modifiable risk factors associated with neonatal, infant and under-five mortalities using nationally representative surveys in LMICs. Our findings revealed that 41.4% of neonatal mortality was attributed to five potentially modifiable risk factors: delayed initiation of breastfeeding, unclean cooking fuel, infrequent ANC visits, maternal lack of formal education, and mother’s lacking two doses of tetanus injections. Similarly, a combination of these five modifiable risk factors contributed to 40.5% of infant deaths. Furthermore, 40.8% of under-five deaths in LMICs were attributed to delayed initiation of breastfeeding, use of unclean cooking fuel, mothers lacking formal education, infrequent ANC visits, and the absence of improved toilet facilities.

The global community has failed to mitigate the disproportionate child mortality rates in LMICs, with an estimated 40 million children projected to lose their lives by 2030 [2,3]. Without immediate intervention, many countries will fall short of the SDG mortality targets by 2030. Achieving the SDG health targets requires an investment of approximately USD 371 billion, excluding costs related to the coronavirus disease 2019 pandemic and the ongoing global conflicts [31]. Allocating resources for breastfeeding, maternal education, high-quality maternal and childcare, and a healthy household environment is evidence-based and can substantially reduce neonatal, infant, and under-five mortality rates in LMICs. Our findings can guide resource allocation, shape public health strategies, and inform policy priorities aimed at reducing early childhood death in LMICs.

Improving children’s survival requires ensuring that women and children have access to quality health care services across the entire spectrum of care, spanning from preconception and pregnancy to delivery, the postnatal period, infancy, and early childhood. However, a significant gap exists in care for women before pregnancy, and relying solely on prenatal care will be inadequate in mitigating obstetric health risks [32]. Ensuring women enter pregnancy in optimal health is crucial, especially considering the high prevalence of unintended pregnancies in LMICs. Addressing this gap, preconception care extends the continuum of maternal care by targeting pre-pregnancy health risks and health conditions.

Establishing well-equipped birthing facilities that provide high-quality care is also crucial to further improve child survival. Prioritising the presence of skilled newborn attendants is equally important to ensure safe delivery and prompt initiation of breastfeeding. Initiating breastfeeding within the first hour of birth is vital to protect the newborn from infection, ensure proper thermal care, positively influence exclusive breastfeeding duration, and stimulate the production of vital colostrum – a crucial source of nutrition and immunity [33]. Additionally, significant investments in training and health infrastructure are imperative to support life-saving interventions, including caesarean delivery and neonatal resuscitation.

To further reduce child mortality, a critical measure involves enhancing home-based care during the postnatal period and beyond. Female community health workers, who reside in the communities they serve, play a pivotal role in delivering various postnatal care services and interventions for lactating women and their newborns [34]. More specifically, community health workers are effective in offering culturally appropriate advice regarding exclusive breastfeeding support for the first six months and promoting a conducive household environment for optimal newborn growth and maternal psychosocial well-being [34]. Additionally, female community health workers can aid in the early diagnosis and treatment of common childhood illnesses, including malaria, pneumonia, and diarrhoea.

The computation of PAFs for neonatal, infant and under-five mortality rates provides an opportunity to guide resource distribution, particularly in countries (e.g. Sierra Leone, Chad, and Pakistan) dealing with a substantial burden of diseases in LMICs. The use of nationally representative DHS data sets further strengthens the applicability of our findings to the broader regional context. The modifiable risk factors investigated in this study hold broader implications for shaping future child health policies.

Despite the above strengths, it is essential to acknowledge certain limitations. First, the use of cross-sectional data impedes the establishment of a clear temporal relationship between the potentially modifiable risk factors and the outcomes. Second, the potential for recall bias should be considered, particularly when assessing modifiable risk factors like the early initiation of breastfeeding. To mitigate this, we focused on the youngest live births within each household, which are more likely to be remembered accurately. However, this approach does not eliminate the risk of bias. To overcome these limitations, future research should employ longitudinal designs to quantify childhood mortalities that will be eliminated by providing breastfeeding.

Third, unmeasured confounders, such as acute respiratory tract infections (ARI) and diarrhoea, could potentially affect the accuracy of the PAF estimates by leading to either overestimation or underestimation. In the DHS, data on ARI and diarrhoea were collected through maternal recall of symptoms occurring within the two weeks before the survey. This method presents inherent challenges for the analysis, as it may not accurately capture the full extent of these conditions, especially considering that many childhood deaths occur in the early years of life, often before symptoms can be reliably recalled. While our study focused on examining population-level modifiable risk factors, which can serve as proxy indicators for common childhood illnesses, it is crucial to acknowledge the limitations posed by these unmeasured confounders. Future longitudinal research is thus recommended to directly examine the impact of ARI and diarrhoea on childhood mortality.

Fourthly, PAF estimates rely on particular assumptions involving causality, the independence of modifiable risk factors, and consistent associations over time [35]. However, these assumptions might prove unrealistic due to the intricate interplay of socio-economic, cultural, health care, maternal, and child-related factors associated with childhood mortality. Despite this complexity, PAFs offer a straightforward and intuitive metric that can supplement other methodologies in pinpointing modifiable risk factors suitable for policy intervention. Finally, while our analysis adjusts for individual-level factors within country-level characteristics, the findings represent broad regional trends across LMICs rather than precise estimates for specific countries. Differences in pregnancy, breastfeeding, feeding practices, and health care systems across countries may influence the effects of modifiable risk factors on child mortality [36]. Despite these variations, our results broadly explain how common risk factors affect neonatal, infant, and under-five mortality. We recommend that future studies focus on how these factors function within distinct health care and cultural contexts.

## CONCLUSIONS

Our study identified five modifiable risk factors that could potentially prevent 40% of neonatal, infant and under-five mortality in LMICs. Given the current global economic climate, we propose that policymakers prioritise these factors when formulating future child health interventions to accelerate the reduction of childhood mortality. Key recommendations include ensuring that women enter pregnancy in optimal health, prioritising the presence of skilled newborn attendants for timely and proper breastfeeding initiation, and enhancing home-based care during the postnatal period and beyond. Commencing in countries with the highest burden of childhood mortality (e.g. Sierra Leone, Chad, and Pakistan) should be the priority.

## Additional material


Online Supplementary Document

## References

[1] United Nations. Transforming our world: The 2030 agenda for sustainable development. New York, New York, USA: United Nations; 2015. Available: https://sustainabledevelopment.un.org/content/documents/21252030%20Agenda%20for%20Sustainable%20Development%20web.pdf. Accessed: 7 January 2025.

[2] United Nations Inter-agency Group for Child Mortality Estimation (UN IGME). Levels and Trends in Child Mortality: Report 2020, estimates developed by the UN Inter-agency Group for Child Mortality Estimation. New York, New York, USA: United Nations Children’s Fund; 2020. Available: https://www.unicef.org/media/60561/file/UN-IGME-child-mortality-report-2019.pdf. Accessed: 7 January 2025.

[3] Sharrow D Hug L You D Alkema L Black R Cousens S Global, regional, and national trends in under-5 mortality between 1990 and 2019 with scenario-based projections until 2030: a systematic analysis by the UN Inter-agency Group for Child Mortality Estimation. Lancet Glob Health. 2022;10:e195–206. 10.1016/S2214-109X(21)00515-5 35063111 PMC8789561

[4] Bai R Dong W Liu J Peng Q Lyu J Trends in Under-5 Mortality Rates and Their Associations with Socioeconomic Factors Among Countries Participating in the Belt and Road Initiative: A Panel Data Analysis. Int J Gen Med. 2021;14:7763–73. 10.2147/IJGM.S332926 34785934 PMC8579877

[5] Kipp AM Blevins M Haley CA Mwinga K Habimana P Shepherd BE Factors associated with declining under-five mortality rates from 2000 to 2013: an ecological analysis of 46 African countries. BMJ Open. 2016;6:e007675. 10.1136/bmjopen-2015-007675 26747029 PMC4716228

[6] Hipgrave DB Alderman KB Anderson I Soto EJ Health sector priority setting at meso-level in lower and middle income countries: Lessons learned, available options and suggested steps. Soc Sci Med. 2014;102:190–200. 10.1016/j.socscimed.2013.11.056 24565157

[7] Sohail H Neupane S Prevalence of and factors associated with under-5 mortality in South Asia. Int Health. 2019;11:119–27. 10.1093/inthealth/ihy065 30285111

[8] Yaya S Bishwajit G Okonofua F Uthman OA Under five mortality patterns and associated maternal risk factors in sub-Saharan Africa: A multi-country analysis. PLoS One. 2018;13:e0205977. 10.1371/journal.pone.0205977 30359408 PMC6201907

[9] Olukade T Uthman OA Caesarean section and increased neonatal mortality risk in meta-analysis of 33 sub-Saharan Africa Demographic and Health Surveys. Acta Paediatr. 2021;110:2780–9. 10.1111/apa.16032 34265122

[10] Ogbo FA Ezeh OK Awosemo AO Ifegwu IK Tan L Jessa E Determinants of trends in neonatal, post-neonatal, infant, child and under-five mortalities in Tanzania from 2004 to 2016. BMC Public Health. 2019;19:1243. 10.1186/s12889-019-7547-x 31500599 PMC6734430

[11] Lin K Chern S Sun J Mapping the quality of prenatal and postnatal care and demographic differences on child mortality in 26 low to middle-income countries. World J Pediatr. 2023;19:835–50. 10.1007/s12519-022-00668-5 36705781

[12] Newson R. PUNAF: Stata module to compute population attributable fractions for cohort studies. 2012. Available: https://ideas.repec.org/c/boc/bocode/s457193.html. Accessed: 7 January 2025.

[13] Newson RB Attributable and unattributable risks and fractions and other scenario comparisons. Stata J. 2013;13:672–98. 10.1177/1536867X1301300402

[14] Northridge ME Public health methods–attributable risk as a link between causality and public health action. Am J Public Health. 1995;85:1202–4. 10.2105/AJPH.85.9.1202 7661224 PMC1615585

[15] Rockhill B Newman B Weinberg C Use and misuse of population attributable fractions. Am J Public Health. 1998;88:15–9. 10.2105/AJPH.88.1.15 9584027 PMC1508384

[16] Poole C A history of the population attributable fraction and related measures. Ann Epidemiol. 2015;25:147–54. 10.1016/j.annepidem.2014.11.015 25721747

[17] Corsi DJ Neuman M Finlay JE Subramanian SV Demographic and health surveys: a profile. Int J Epidemiol. 2012;41:1602–13. 10.1093/ije/dys184 23148108

[18] United States Agency for International Development. Guide to DHS Statistics: DHS-7. Rockville, Maryland, USA: United States Agency for International Development; 2018. Available: https://dhsprogram.com/pubs/pdf/DHSG1/Guide_to_DHS_Statistics_DHS-7.pdf Accessed: 7 January 2025.

[19] Ahmed KY Agho KE Page A Arora A Ogbo FA Mapping Geographical Differences and Examining the Determinants of Childhood Stunting in Ethiopia: A Bayesian Geostatistical Analysis. Nutrients. 2021;13:2104. 10.3390/nu13062104 34205375 PMC8234472

[20] Ahmed KY Page A Arora A Ogbo FA Associations between infant and young child feeding practices and acute respiratory infection and diarrhoea in Ethiopia: A propensity score matching approach. PLoS One. 2020;15:e0230978. 10.1371/journal.pone.0230978 32236145 PMC7112197

[21] Ogbo FA Page A Idoko J Agho KE Population attributable risk of key modifiable risk factors associated with non-exclusive breastfeeding in Nigeria. BMC Public Health. 2018;18:247. 10.1186/s12889-018-5145-y 29439701 PMC5812198

[22] Ahmed KY Dadi AF Ogbo FA Page A Agho KE Akalu TY Population-Modifiable Risk Factors Associated With Childhood Stunting in Sub-Saharan Africa. JAMA Netw Open. 2023;6:e2338321. 10.1001/jamanetworkopen.2023.38321 37851439 PMC10585405

[23] George A Stead TS Ganti L What’s the Risk: Differentiating Risk Ratios, Odds Ratios, and Hazard Ratios? Cureus. 2020;12:e10047. 10.7759/cureus.10047 32983737 PMC7515812

[24] Leyland AH Groenewegen PP Multilevel modelling and public health policy. Scand J Public Health. 2003;31:267–74. 10.1080/14034940210165028 15099032

[25] Peugh JL A practical guide to multilevel modeling. J Sch Psychol. 2010;48:85–112. 10.1016/j.jsp.2009.09.002 20006989

[26] Miettinen OS Proportion of disease caused or prevented by a given exposure, trait or intervention. Am J Epidemiol. 1974;99:325–32. 10.1093/oxfordjournals.aje.a121617 4825599

[27] Wilson LF Page AN Dunn NA Pandeya N Protani MM Taylor RJ Population attributable risk of modifiable risk factors associated with invasive breast cancer in women aged 45–69 years in Queensland, Australia. Maturitas. 2013;76:370–6. 10.1016/j.maturitas.2013.09.002 24113278

[28] See RS Thompson F Russell S Quigley R Esterman A Harriss LR Potentially modifiable dementia risk factors in all Australians and within population groups: an analysis using cross-sectional survey data. Lancet Public Health. 2023;8:e717–25. 10.1016/S2468-2667(23)00146-9 37633680

[29] StataCorp LLC. Stata survey data reference manual release 15. College Station, Texas, USA: Stata Press Publication StataCorp LLC; 2017. Available: https://surveydesign.com.au/docs/manuals/stata15/svy.pdf. Accessed: 7 January 2025.

[30] Rabe-Hesketh S. GLLAMM: Stata program to fit generalised linear latent and mixed models. Berkeley, California, USA: University of California; 2000. Available: https://biostat.jhsph.edu/~fdominic/teaching/bio656/software/gllamm.manual.pdf. Accessed: 7 January 2025.

[31] Economist Impact. Breaking the cycle of chronic child malnutrition in Sub-Saharan Africa. London, UK: The Economist Group; 2023. Available: https://impact.economist.com/perspectives/sites/default/files/economist_impact_breaking_the_cycle_of_chronic_malnutrition_in_ssa_feb_23.pdf. Accessed: 7 January 2025.

[32] Dean SV Lassi ZS Imam AM Bhutta ZA Preconception care: closing the gap in the continuum of care to accelerate improvements in maternal, newborn and child health. Reprod Health. 2014;11:S1. 10.1186/1742-4755-11-S3-S1 25414942 PMC4196556

[33] World Health Organization. Capture the moment - Early initiation of breastfeeding: the best start for every newborn. Geneva, Switzerland: World Health Organization; 2018. Available: https://www.who.int/nutrition/publications/infantfeeding/capture-moment-early-initiation-bf/en/. Accessed: 3 April 2023.

[34] Aboubaker S Qazi S Wolfheim C Oyegoke A Bahl R Community health workers: A crucial role in newborn health care and survival. J Glob Health. 2014;4:020302. 10.7189/jogh.04.020302 25520788 PMC4267086

[35] Eide GE Attributable fractions for partitioning risk and evaluating disease prevention: a practical guide. Clin Respir J. 2008;2:92–103. 10.1111/j.1752-699X.2008.00091.x 20298357

[36] Zong X Wu H Zhao M Magnussen CG Xi B Global prevalence of WHO infant feeding practices in 57 LMICs in 2010–2018 and time trends since 2000 for 44 LMICs. EClinicalMedicine. 2021;37:100971. 10.1016/j.eclinm.2021.100971 34386748 PMC8343261

