# Supplementary material for: Population attributable fractions for modifiable risk factors of neonatal, infant, and under-five mortality in 48 low- and middle-income countries
Source: J Glob Health. 2025 Jan 17;15:04015. doi: 10.7189/jogh.15.04015 (PMC11739818; doi:10.7189/jogh.15.04015)
Supplement: Online Supplementary Document [file jogh-15-04015-s001.pdf]

Supplement to: Ahmed KY, Thapa S, Kibret GD, Bizuayehu HM, Sun J, Huda MM, Dadi AF, Ogbo FA, Mahmood S, Shiddiky MJA, Tadese F, Aychiluhm SB, Anyasodor AE, Ross AG. Population attributable fractions for modifiable risk factors of neonatal, infant, and under-five mortality in 48 low- and middle-income countries. J Glob Health. 2025;15:04015.

Table S1: List of countries and sample size included

List of countries	Weighted sample	Response rates for women's questionnaire
Afghanistan	19422	96.8%
Angola	8274	94.2%
Bangladesh	7493	98.8%
Benin	8724	97.6%
Burkina Faso	8972	98.3%
Burundi	8763	97.6%
Cambodia	6686	98.2%
Cameroon	6407	98.0%
Chad	10704	92.1%
Comoros	1821	93.2%
Congo (Brazzaville)	5729	98.0%
Congo Democratic Republic	10596	98.6%
Côte d'Ivoire	5039	98.1%
Ethiopia	7343	94.6%
Gabon	4255	95.9%
The Gambia	5234	95.1%
Ghana	4022	97.3%
Guinea	5278	99.0%
India	172318	96.9%
Indonesia	14914	97.8%
Kenya	13292	96.6%
Kyrgyzstan	2976	99.1%
Lesotho	2513	97.1%
Liberia	3922	96.4%
Madagascar	9072	94.9%
Malawi	13103	97.7%
Maldives	2328	84.0%
Mali	6425	97.6%
Mauritania	7475	96.2%
Mozambique	7415	99.8%
Myanmar	3547	95.8%
Namibia	3756	92.3%
Nepal	4207	97.4%
Niger	7650	95.4%
Nigeria	21370	99.3%
Pakistan	6601	93.2%
Philippines	6432	98.0%
Rwanda	6167	99.7%
Senegal	3958	96.1%
Sierra Leone	7087	96.7%
South Africa	2968	86.2%
Tajikistan	4320	99.2%
Tanzania	6891	97.3%
Timor-Leste	4929	97.0%
Togo	4692	97.8%
Uganda	9915	97.0%
Zambia	7145	96.4%
Zimbabwe	4841	96.2%

Appendix S2: Variables measurement

Modifiable risk factors

The modifiable risk factors were broadly categorized into three groups: child factors (perceived baby birth size and early initiation of breastfeeding), maternal factors (maternal education, maternal employment, antenatal care (ANC) visits, maternal tetanus toxoid vaccination, and place of birth) and household factors (household wealth index, type of toilet system, source of drinking water, and type of cooking fuel). This classification is based on previously published studies in LMICs[1-4].

Perceived birth size

The size of the child at birth is classified as small or very small, and average or larger, and is based on the mother's report of the relative size of the child at birth. For this study, we grouped small or very small as "*below average*" and average or larger as "*average and above birth size*", based on previously published study[5].

Early initiation of breastfeeding

The initiation of breastfeeding indicators was reported for all children born in the 5 years before the survey. We calculated early initiation of breastfeeding (EIBF) as a percentage of children who started breastfeeding within one hour of birth. For this study, EIBF was grouped as '1' = '*initiated breastfeeding within 1 h of birth*', or '2' = '*Not initiated breastfeeding within 1 h of birth*', based on the previously published studies[5,6].

Maternal education

In DHS, maternal education is generally reported as the highest level of education attended (not necessarily completed) (in categories of no education, primary, secondary, higher than secondary. For this study, we regrouped maternal education as '1' = '*no or low schooling*' or '3' = '*secondary education or higher*'.

ANC visits and place of birth

The number of antenatal visits is grouped into categories of 'no or low antenatal care visits' and 4+ visits, and place of birth was grouped as *home* or *health facility*.

Wealth Index

The wealth index represents a combined measurement of a household's overall living standards. It is determined by assessing various factors, including the household's ownership of specific assets like televisions and cars, dwelling features such as flooring material, drinking water source, and toilet facilities. Each asset's importance is calculated using factor scores derived from principal components analysis (PCA). These scores are standardized to have a mean of zero and a standard deviation of one. Based on whether a household owns a particular asset, standardized scores are assigned and summed up. Individuals are then ranked according to their household's total score. Detailed procedures for wealth index construction are available elsewhere[7].

The population is divided into five equal groups, or quintiles, to establish wealth categories: Lowest, Second, Middle, Fourth, and Highest. For this study, the household wealth index was regrouped as '1' = 'poor or medium households', '2' = 'rich households (fourth and highest quintile)', based on previously published studies[5,6,8-10].

Type of cooking fuel

The study also considered the type of cooking fuel among modifiable risk factors. For this study, households that used electricity, natural gas, biogas, or kerosene as a cooking fuel were classified as '*clean*', while those households that used charcoal, firewood, or dung were grouped as '*not clean*'. This classification is based on previously published studies[11,12].

Source of drinking water

The source of drinking water and type of toilet facility w ere classified as ‘improved’ or ‘not improved’, based on the taxonomy of the WHO and UNICEF Joint Monitoring Programme (JMP) for Water and Sanitation[13] as applied in past studies[4,6]. Households that used piped water, public tap or standpipe, a tube well or borehole, a protected well/spring, rainwater and/or bottled water were classified as ‘*improved’*. Households that used unprotected well/spring, tanker truck/cart, surface water, and/or sachet water were grouped as ‘*not improved*’[13].

Type of toilet system

The type of toilet facility was also grouped as ‘*improved’* (included flush/pour-flush toilets or flush/pour-flush toilets piped to the sewer system, septic tank or pit latrine; ventilated improved pit (VIP) latrine; pit latrine with slab and/or composting toilet). ‘*Not improved*’ type of facility included flush/pour-flush not piped to sewer, septic tank or pit latrine; pit latrine without slab/open pit; bucket or hanging toilet/hanging latrine and no facility/bush/field[13].

Appendix S3: Statistical analysis

Step 1: descriptive statistics

Frequencies and percentages were calculated to provide an overview of the study population and the prevalence of neonatal, infant and under-five mortality across the study factors. All descriptive analyses accounted for the sampling weights, clustering, and stratification using the 'svy' command in STATA.

Step 2: Generalised linear latent and mixed models

Variable selection

The modifiable factors were selected based on past literature[1-4] their importance for the outcomes, availability of data, and the amenability for policy interventions in improving child health and survival. In this study, maternal BMI was excluded due to the missing maternal BMI for some countries (e.g., Angola).

Our selection of covariates was based on: 1) previously published studies[3,4], (ii) by excluding potential mediators (variables with a potential causal link between modifiable risk factors and outcomes), and (iii) their statistical significance with the outcome. In our analysis, we initially considered the gender of the baby, birth order, maternal age, family size and place of residence as potential covariates, as they were less likely to be part of the causal pathway. However, in the final model, only place of residence and family size retained the significant associations with the outcome.

The Generalised Linear Latent and Mixed Models (GLLAMM) were used to determine the odds ratios (ORs) and 95% confidence intervals for modifiable risk factors of neonatal, infant and under-five mortality. Our GLLAMM models were structured in two levels, individual (e.g., child, maternal and household factors) and community levels (place of residence) to account for the hierarchical nature of the data, wherein children under five years old are nested within geographic clusters.

Multilevel modelling offers distinct advantages compared to classical single-level logistic regression models. Firstly, it acknowledges the hierarchical nature of data, recognizing that children under-five (level I) is nested within clusters (level II). Failure to account for these hierarchies results in underestimated standard errors of regression coefficients, leading to an overstatement of statistical significance. Secondly, multilevel modelling addresses the dependence of observations within the same clusters; children within the same cluster tend to be more similar than those in different clusters. Lastly, it allows for the simultaneous estimation of cluster-level effects (random effects) and the assessment of associations for community-level predictors, such as place of residence.

The multilevel models were constructed in three steps. Initially, a null unconditional model was developed in stage one, without any study variable. In stage two, individual-level factors (including child, maternal, and household factors) were incorporated into the model. Stage three introduced community-level factors and presented in the results, encompassed both individual and community-level factors. This final model, which included both individual and community-level factors, was chosen due to its minimal deviance and superior ability to explain the variation in the outcome variables.

Step 3: Population-attributable fractions

PAF quantifies the percentage of neonatal mortality in SSA that could potentially be averted by addressing the identified modifiable risk factors among the populations[14]. Once the modifiable risk factors for neonatal, infant, and under-five mortalities were identified in the GLLAMM analysis, we calculated the PAFs using Miettinen's formula. The choice of Miettinen's formula was based on its ability to provide valid estimates even in the presence of confounding, particularly when using adjusted RRs[15,16].

$$\boldsymbol{PAF = Pc(OR - 1)/\,OR}$$

Where Pc is the prevalence of the modifiable risk factor among cases, and OR is the adjusted ORs of neonatal, infant, and under-five mortality associated with the modifiable risk factors. Based on previously published studies[17], we employed communality weights to correct for the overlap of risk factors among participants[17].

Initially, we computed the pairwise tetrachoric correlation between all potential modifiable risk factors. Subsequently, a principal components analysis was conducted on the tetrachoric correlation matrix. The communality for each risk factor was determined by the sum of squares of the loadings in all principal components with an eigenvector greater than 1. The weighting of each risk factor was then carried out using the formula: We = 1 − communality.

Commmunality calculations

**variables**	**Comp1**	**Comp2**	**Comp3**	**Square1**	**Square2**	**Square3**	**Communalities (Sum of squares)**	**weight**
Perceived birth size	0.08616	0.3189	0.3779	0.007424	0.101697	0.142808	0.251929166	0.748071
early initiation of breastfeeding	0.06734	0.3151	0.7251	0.004535	0.099288	0.52577	0.629592696	0.370407
maternal education	0.4051	-0.03961	-0.1405	0.164106	0.001569	0.01974	0.185415212	0.814585
antenatal care visits	0.3255	0.4453	-0.2427	0.10595	0.198292	0.058903	0.36314563	0.636854
place of birth	0.4031	0.2207	0.08611	0.16249	0.048708	0.007415	0.218613032	0.781387
wealth status	0.416	-0.3082	0.07936	0.173056	0.094987	0.006298	0.27434125	0.725659

toilet facilities	0.395	-0.2174	0.1979	0.156025	0.047263	0.039164	0.24245217	0.757548
source of drinking water	0.2459	-0.2084	0.1526	0.060467	0.043431	0.023287	0.12718413	0.872816
cooking fuel	0.3579	-0.291	-0.1458	0.128092	0.084681	0.021258	0.23403105	0.765969
Tetanus vaccination	0.1946	0.5293	-0.3941	0.037869	0.280158	0.155315	0.47334246	0.526658

Following this, a combined PAF across the modifiable risk factors was calculated using the specified formula:

$$\boldsymbol{PAF\ (combined) = 1 - \prod_{r=1}^{R}(1 - WePAFe)}$$

Where '*e*' represents each modifiable risk factor, and 'We' represents the communality weight of ear risk factor. Finally, we estimated the adjusted PAF for each individual risk factor using the formula:

$$\boldsymbol{adjusted\ PAFe = ([PAFe / \sum PAFe] * combined\ PAF.}$$

Step 4: Checking assumptions

We checked our model estimation for the normal distribution of Random Effects and scatter plot of residuals against fitted values.

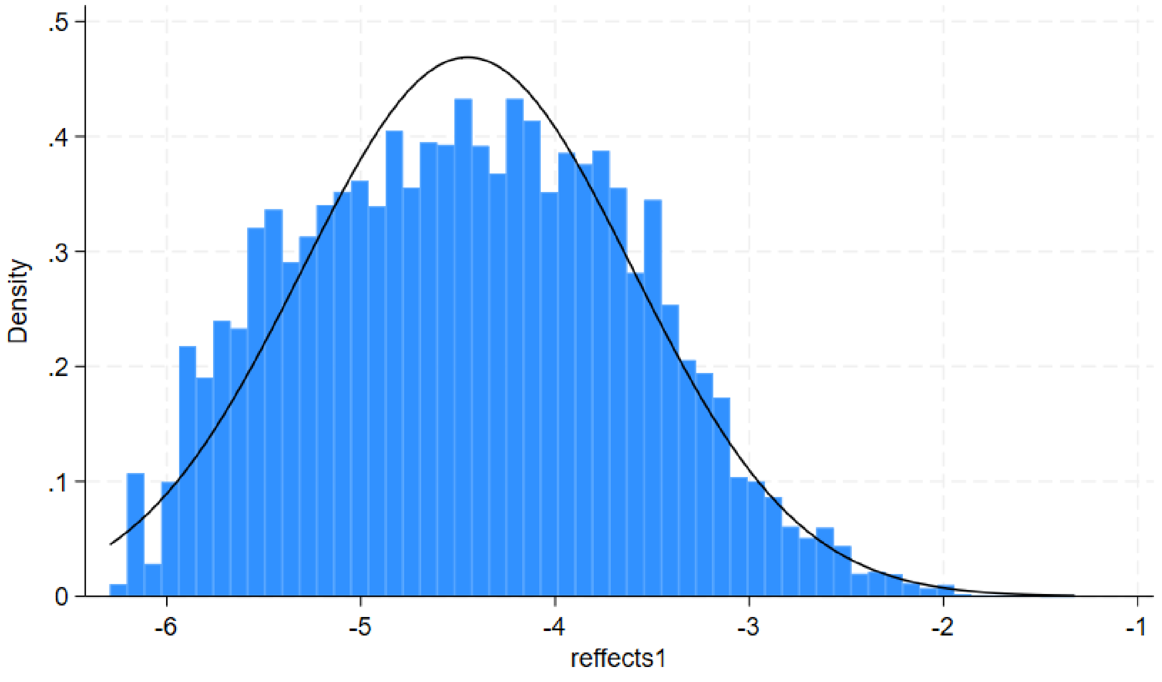

Figure 1: Histogram showing the normal distribution of random effects for neonatal mortality

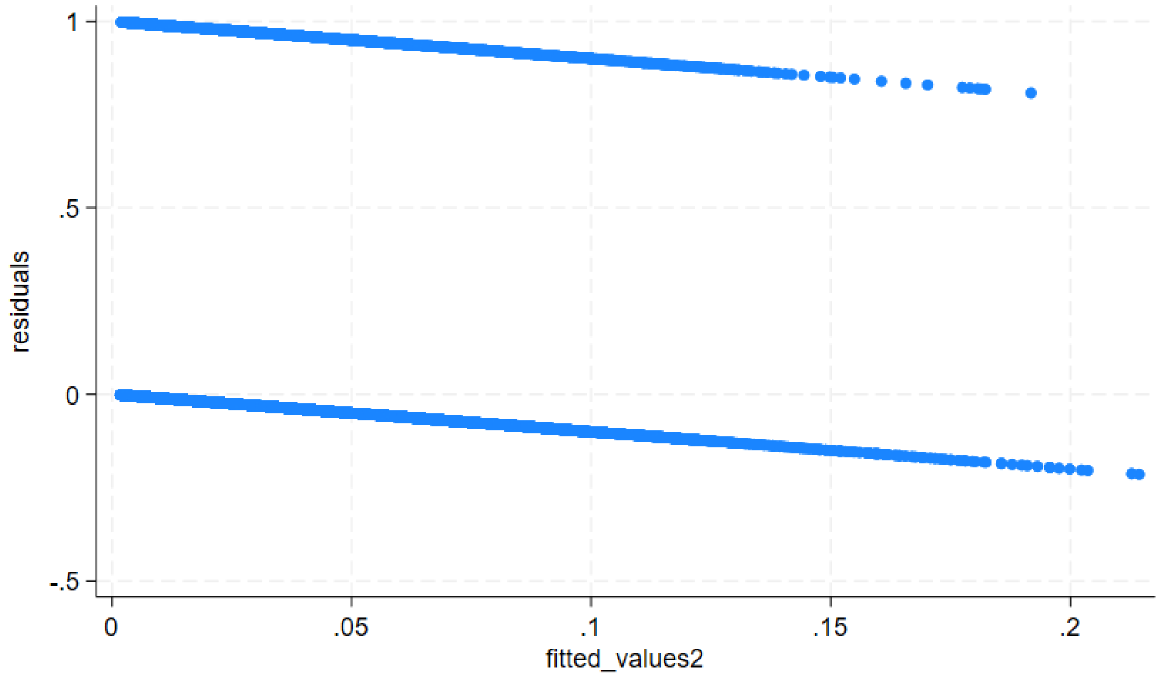

Figure 2: Scatter plot of residuals against fitted values for neonatal mortality

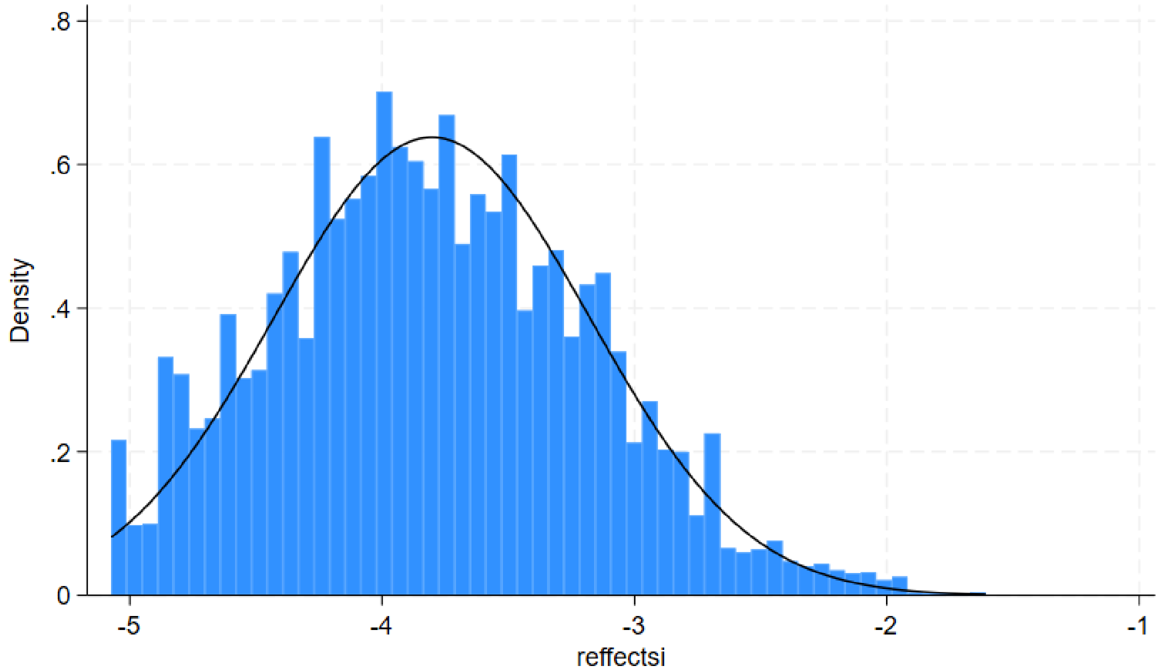

Figure 3: Histogram showing the normal distribution of random effects for infant mortality

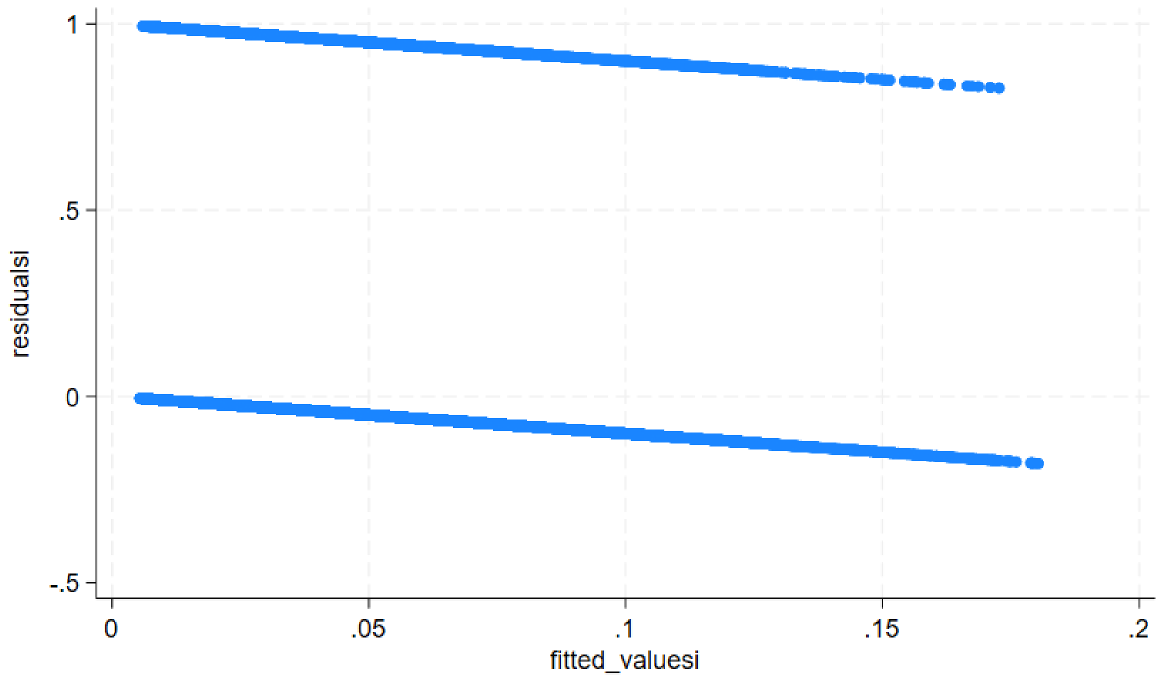

Figure 4: Scatter plot of residuals against fitted values for infant mortality

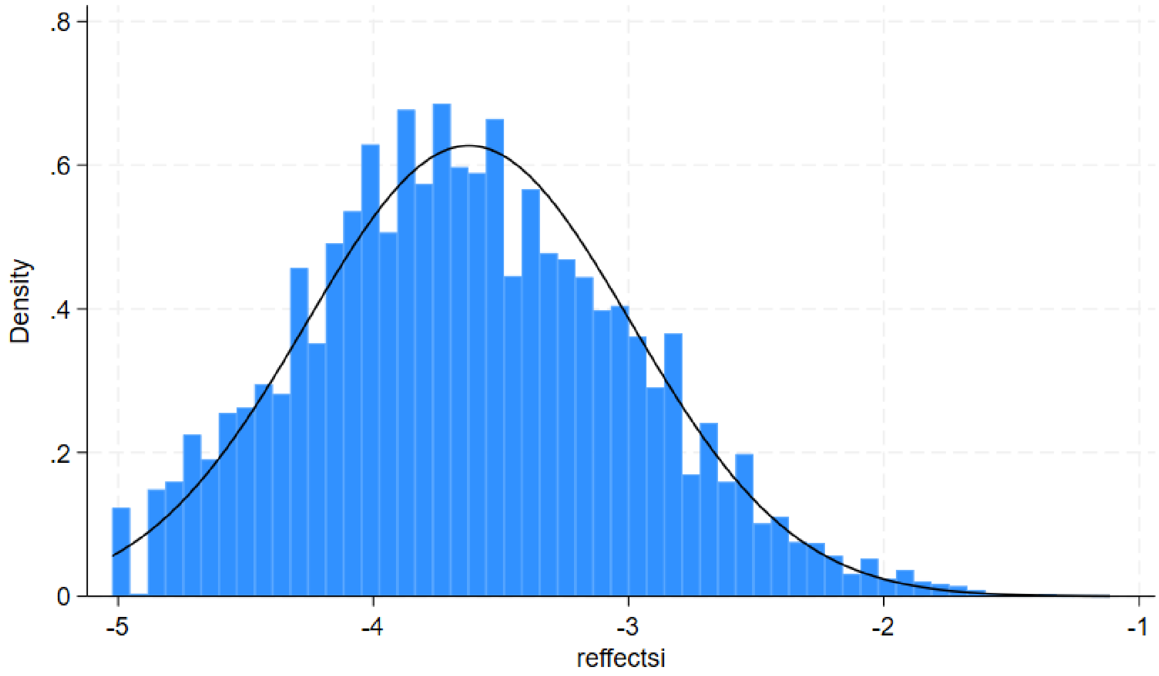

Figure 5: Histogram showing the normal distribution of random effects for under five mortality

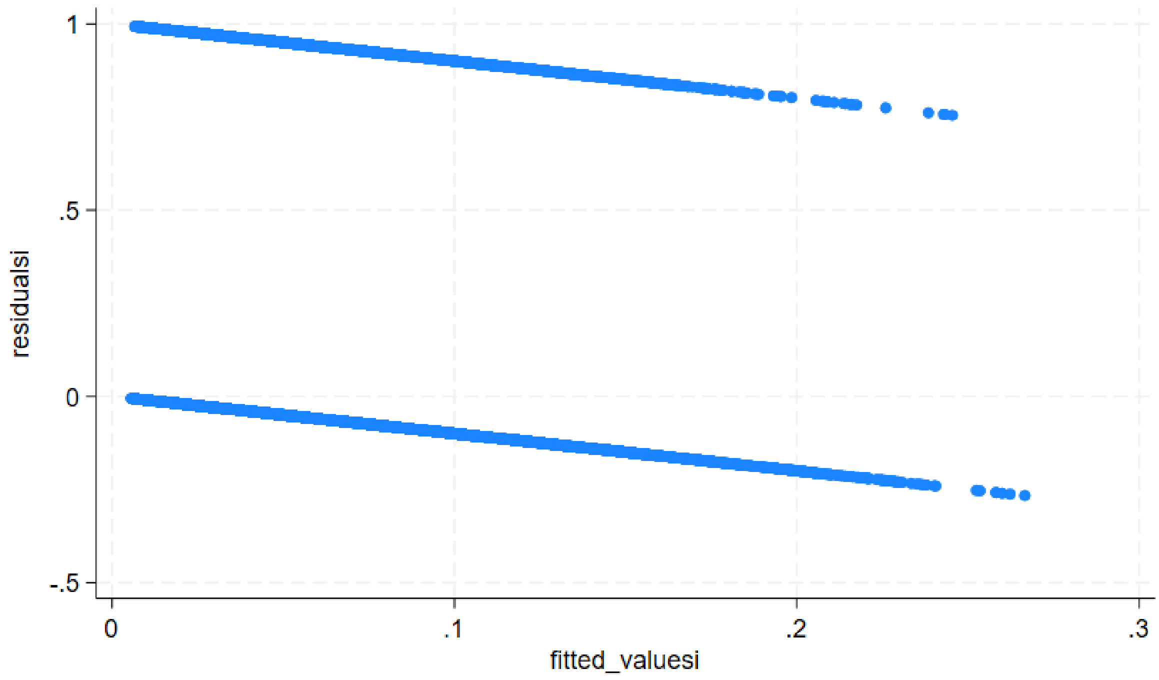

Figure 6: Scatter plot of residuals against fitted values for under five mortality

Finally, to address potential imbalances and unequal probabilities in household selections, non-responses, and to account for clustering and stratification, we applied survey weighting to the data using the 'svy' command in STATA (version 15.0, Stata Corp, College Station, TX, USA)[18]. The regression analysis was conducted using the 'GLLAMM' package for STATA[19]. The association between the modifiable risk factors and the outcome variables was presented in terms of RRs along with 95% CIs.

Appendix 4: DHS data access grant letter

Oct 24, 2022

Kedir Yimam Ahmed
Samara University
Ethiopia
Request Date: 10/24/2022

Dear Kedir Yimam Ahmed:

This is to confirm that you are approved to use the following Survey Datasets for your registered research paper titled: "Determinants of childhood stunting in Sub-Saharan Africa ":

> **Angola, Benin, Botswana, Burkina Faso, Burundi, Cameroon, Central African Republic, Chad, Comoros, Congo, Congo Democratic Republic, Cote d'Ivoire, Eritrea, Eswatini, Ethiopia, Gabon, Gambia, Ghana, Guinea, Kenya, Lesotho, Liberia, Madagascar, Malawi, Mali, Mauritania, Mozambique, Namibia, Niger, Nigeria, Nigeria (Ondo State), Rwanda, Sao Tome and Principe, Senegal, Sierra Leone, South Africa, Sudan, Tanzania, Togo, Uganda, Zambia, Zimbabwe**

For restricted surveys, you must also request special permission from the Implementing Agencies. If approved, the restricted datasets will be provided to you by FTP.

To access the datasets, please login at: https://www.dhsprogram.com/data/dataset_admin/login_main.cfm. The user name is the registered email address, and the password is the one selected during registration.

The IRB-approved procedures for DHS public-use datasets do not in any way allow respondents, households, or sample communities to be identified. There are no names of individuals or household addresses in the data files. The geographic identifiers only go down to the regional level (where regions are typically very large geographical areas encompassing several states/provinces). Each enumeration area (Primary Sampling Unit) has a PSU number in the data file, but the PSU numbers do not have any labels to indicate their names or locations. In surveys that collect GIS coordinates in the field, the coordinates are only for the enumeration area (EA) as a whole, and not for individual households, and the measured coordinates are randomly displaced within a large geographic area so that specific enumeration areas cannot be identified.

The DHS Data may be used only for the purpose of statistical reporting and analysis, and only for your registered research. To use the data for another purpose, a new research project must be registered. All DHS data should be treated as confidential, and no effort should be made to identify any household or individual respondent interviewed in the survey. Also, be aware that re-distribution of any DHS micro-level data, either directly or within any tool/dashboard, is not permitted. Please reference the complete terms of use at: https://dhsprogram.com/Data/terms-of-use.cfm.

The data must not be passed on to other researchers without the written consent of DHS. However, if you have coresearchers registered in your account for this research paper, you are authorized to share the data with them. All data users are required to submit an electronic copy (pdf) of any reports/publications resulting from using the DHS data files to: references@dhsprogram.com.

Sincerely,

Bridgette Wellington

Bridgette Wellington
Data Archivist
The Demographic and Health Surveys (DHS) Program

530 Gaither Road, Suite 500, Rockville, MD 20850 USA +1.301.407.6500 +1.301.407.6501 fax icf.com

References

1. Ahmed KY, Agho KE, Page A, Arora A, Ogbo FA. Mapping Geographical Differences and Examining the Determinants of Childhood Stunting in Ethiopia: A Bayesian Geostatistical Analysis. *Nutrients* 2021; **13**(6).
2. Ahmed KY, Page A, Arora A, Ogbo FA. Associations between infant and young child feeding practices and acute respiratory infection and diarrhoea in Ethiopia: A propensity score matching approach. *PloS one* 2020; **15**(4): e0230978.
3. Ogbo FA, Page A, Idoko J, Agho KE. Population attributable risk of key modifiable risk factors associated with non-exclusive breastfeeding in Nigeria. *BMC public health* 2018; **18**(1): 247.
4. Ahmed KY, Dadi AF, Ogbo FA, et al. Population-Modifiable Risk Factors Associated With Childhood Stunting in Sub-Saharan Africa. *JAMA Network Open* 2023; **6**(10): e2338321-e.
5. Ahmed KY, Agho KE, Page A, et al. Mapping Geographical Differences and Examining the Determinants of Childhood Stunting in Ethiopia: A Bayesian Geostatistical Analysis. *Nutrients* 2021; **13**(6).
6. Ahmed KY, Page A, Arora A, Ogbo FA, Global M, Child Health Research c. Associations between infant and young child feeding practices and acute respiratory infection and diarrhoea in Ethiopia: A propensity score matching approach. *PloS one* 2020; **15**(4): e0230978.
7. The DHS Program. Wealth Index Construction. 2016. https://www.dhsprogram.com/topics/wealth-index/Wealth-Index-Construction.cfm.
8. Ahmed KY, Abrha S, Page A, et al. Trends and determinants of underweight and overweight/obesity among urban Ethiopian women from 2000 to 2016. *BMC public health* 2020; **20**(1): 1276.
9. Ahmed KY, Ross AG, Hussien SM, Agho KE, Olusanya BO, Ogbo FA. Mapping Local Variations and the Determinants of Childhood Stunting in Nigeria. *International journal of environmental research and public health*, 2023. (accessed.
10. Ahmed KY, Rwabilimbo AG, Abrha S, et al. Factors associated with underweight, overweight, and obesity in reproductive age Tanzanian women. *PloS one* 2020; **15**(8): e0237720.
11. Naz S, Page A, Agho KE. Household Air Pollution and Under-Five Mortality in Bangladesh (2004-2011). *International journal of environmental research and public health* 2015; **12**(10): 12847-62.
12. Naz S, Page A, Agho KE. Household air pollution from use of cooking fuel and under-five mortality: The role of breastfeeding status and kitchen location in Pakistan. *PloS one* 2017; **12**(3): e0173256-e.
13. Girma M, Hussein A, Norris T, et al. Progress in Water, Sanitation and Hygiene (WASH) coverage and potential contribution to the decline in diarrhea and stunting in Ethiopia. *Maternal & child nutrition* 2021: 1.
14. Miettinen OS. Proportion of disease caused or prevented by a given exposure, trait or intervention. *American journal of epidemiology* 1974; **99**(5): 325-32.
15. Ahmad K, Rasmus Oestergaard N, Mohammad Ali M. Methods matter: population attributable fraction (PAF) in sport and exercise medicine. *British Journal of Sports Medicine* 2020; **54**(17): 1049.
16. Khosravi A, Nazemipour M, Shinozaki T, Mansournia MA. Population attributable fraction in textbooks: Time to revise. *Global Epidemiology* 2021; **3**: 100062.
17. Sue See R, Thompson F, Russell S, et al. Potentially modifiable dementia risk factors in all Australians and within population groups: an analysis using cross-sectional survey data. *The Lancet Public Health* 2023; **8**(9): e717-e25.
18. StataCorp. Stata survey data reference manual release 15. College Station, Texas A Stata Press Publication StataCorp LLC; 2017.
19. Rabe-Hesketh S. GLLAMM: Stata program to fit generalised linear latent and mixed models. 2000.
